# A host-encoded prophage targets a Candidate Phyla Radiation bacterium and shapes episymbiotic interactions

**DOI:** 10.1073/pnas.2615890123

**Published:** 2026-09-08

**Authors:** Ajay Kumar, Nusrat Nahar, Deepak Chouhan, Jeffrey S. McLean, Batbileg Bor, Pu-Ting Dong, Xuesong He

**Affiliations:** ^a^ Department of Microbiology, American Dental Association Forsyth Institute, Somerville, MA 02143; ^b^ Department of Periodontics, University of Washington, Seattle, WA 98195; ^c^ Department of Oral Health Sciences, University of Washington, Seattle, WA 98195; ^d^ Department of Microbiology, University of Washington, Seattle, WA 98195; ^e^ Department of Biomedical Engineering, Stony Brook University, Stony Brook, NY 11794; ^f^ Department of Microbiology and Immunology, Stony Brook University, Stony Brook, NY 11794

**Keywords:** Patescibacteria, bacteriophage, episymbiosis, oral microbiome, Candidate Phyla Radiation

## Abstract

The Patescibacteriota, also known as the Candidate Phyla Radiation (CPR), represent a large lineage of ultrasmall bacteria with highly reduced genomes and obligate dependence on bacterial hosts. Although genomic analyses have revealed CRISPR–Cas and restriction–modification systems in many CPR genomes, no cognate bacteriophages (phages) have been isolated, leaving CPR–phage interactions unexplored. *Nanosynbacter lyticus* TM7x, the first cultivated CPR bacterium, grows episymbiotically on its host, *Schaalia odontolytica* XH001, in the human oral microbiome. Here, we identify Xhp1, an inducible prophage of XH001 that is preferentially activated during episymbiosis with TM7x. Released Xhp1 particles infect prophage-free XH001 via distinct strategies determined by host growth mode, establishing lysogeny under planktonic conditions but driving lytic infection during surface-associated growth. Xhp1 also binds efficiently to TM7x and exhibits limited infection under the conditions tested, indicating direct phage–CPR interactions. Importantly, TM7x modulates Xhp1 availability in a spatially dependent manner. In planktonic culture, free-floating TM7x reduces lysogenic conversion of XH001ΔXhp1, consistent with TM7x acting as a phage sink that lowers effective phage concentration. In contrast, during surface-associated growth, TM7x increases XH001ΔXhp1 susceptibility to lytic infection, likely by locally concentrating phage particles within a constrained niche. These results demonstrate that CPR bacteria can regulate viral encounter rates through spatial organization. In spatially structured environments such as oral biofilms, such modulation may shape infection dynamics and community structure. Together, this work characterizes the first CPR-targeting phage and reveals a an important role for phages in CPR–host bacteria interactions.

## Significance

Candidate Phyla Radiation (CPR) bacteria constitute a major fraction of microbial diversity, yet their interactions with bacteriophages (phages) have remained largely speculative due to a lack of experimentally tractable systems. Using the first cultivated CPR strain, *Nanosynbacter lyticus* TM7x, and its host bacterium, we show that a host-encoded prophage is activated during episymbiosis and can directly target TM7x. This finding demonstrates that CPR bacteria are integrated into viral infection networks and that phages can directly modulate CPR–host relationships. Our findings reveal phages as previously unrecognized regulators of CPR ecology and highlight their roles in shaping symbiotic interactions within complex microbial communities.

The Patescibacteriota, also known as the Candidate Phyla Radiation (CPR), comprise an exceptionally large and phylogenetically diverse bacterial superphylum that accounts for more than 25% of known microbial diversity (1–3). CPR bacteria are distinguished by ultrasmall cell sizes, highly reduced genomes, and limited metabolic capacity, features that underlie their obligate dependence on other bacteria for survival (3–7). Rather than functioning as independent metabolic units, many CPR lineages persist through intimate physical associations with bacterial hosts, positioning them as pervasive yet cryptic modulators of microbial community structure and function (6–8).

Despite their reduced genomes, comparative genomic and metagenomic analyses have revealed that many CPR bacteria encode antiviral defense systems, including CRISPR–Cas and restriction–modification modules (9–13). These observations suggest that CPR organisms, like free-living bacteria, experience sustained selective pressure from bacteriophages (phages) and participate in viral arms races within microbial ecosystems. However, despite extensive indirect evidence for CPR–phage interactions (9, 10, 12, 14), no phages that directly target CPR bacteria have been isolated or experimentally characterized to date. Consequently, the identity of CPR-infecting phages, their infection strategies, and their ecological roles in shaping CPR–host associations remain largely unknown. This gap has limited our understanding of how viral predation influences symbiotic stability and population dynamics in CPR-containing microbial communities.

*Nanosynbacter lyticus* strain TM7x is the first cultivated CPR bacterium and was coisolated from the human oral cavity together with its bacterial host, *Schaalia odontolytica* (formerly *Actinomyces odontolyticus*) strain XH001 (6, 15–17). TM7x forms an obligate episymbiotic association, growing on the surface of XH001 cells and relying on the host for essential cellular resources (6, 15, 16). This close physical coupling, combined with experimental accessibility, has established the TM7x–XH001 partnership as a powerful model for studying CPR–host symbioses and their broader ecological impacts within complex microbial environments such as the oral microbiome (18–20).

Notably, *S. odontolytica* strain XH001 harbors an inducible prophage, Xhp1 (6, 16, 21, 22). Prophages are increasingly appreciated as key ecological regulators that influence bacterial fitness, stress responses, and species interactions, rather than merely dormant genetic elements (23–27). In densely structured microbial communities, prophage induction can alter local viral loads, reshape competitive dynamics, and modulate interactions between neighboring cells (26). Given the spatial intimacy of the TM7x–XH001 episymbiosis relationship, the coexistence of a CPR symbiont and an inducible prophage within the same microscale niche raises the possibility that prophage activity both responds to and reshapes host–symbiont interactions.

In this study, we demonstrate that the prophage Xhp1 is preferentially induced during episymbiosis between XH001 and TM7x. Unexpectedly, we find that released Xhp1 particles can bind to and infect TM7x cells, providing direct experimental evidence for a phage targeting a CPR bacterium. This interaction establishes a naturally occurring, experimentally accessible system in which a host-encoded prophage links bacterial and CPR populations within a shared ecological context. Our findings reveal that phages can directly participate in CPR biology and act as modulators of CPR–host interaction dynamics. Together, these results uncover a previously unrecognized ecological role for phages in CPR-containing ecosystems and highlight phages as integral components of multilevel interaction networks that shape microbial community organization.

## Results

### TM7x Association Promotes Induction of the XH001-Encoded Prophage Xhp1.

*N. lyticus* TM7x, a Saccharibacterial species belonging to CPR, forms an obligate episymbiotic association with its bacterial host *S. odontolytica* XH001 in the human oral cavity (6). The resulting coculture (XH001/TM7x) is heterogeneous, comprising TM7x-attached and TM7x-free XH001 subpopulations, as well as abundant free-floating TM7x cells (8, 28). Previous work demonstrated that XH001 harbors an inducible prophage, Xhp1, which enhances XH001 biofilm formation (22). Because TM7x association is known to elicit stress responses in XH001 (6) and cellular stress is a well-established trigger for prophage induction (27, 29), we investigated whether TM7x episymbiosis promotes activation of Xhp1.

**Fig. 1.**
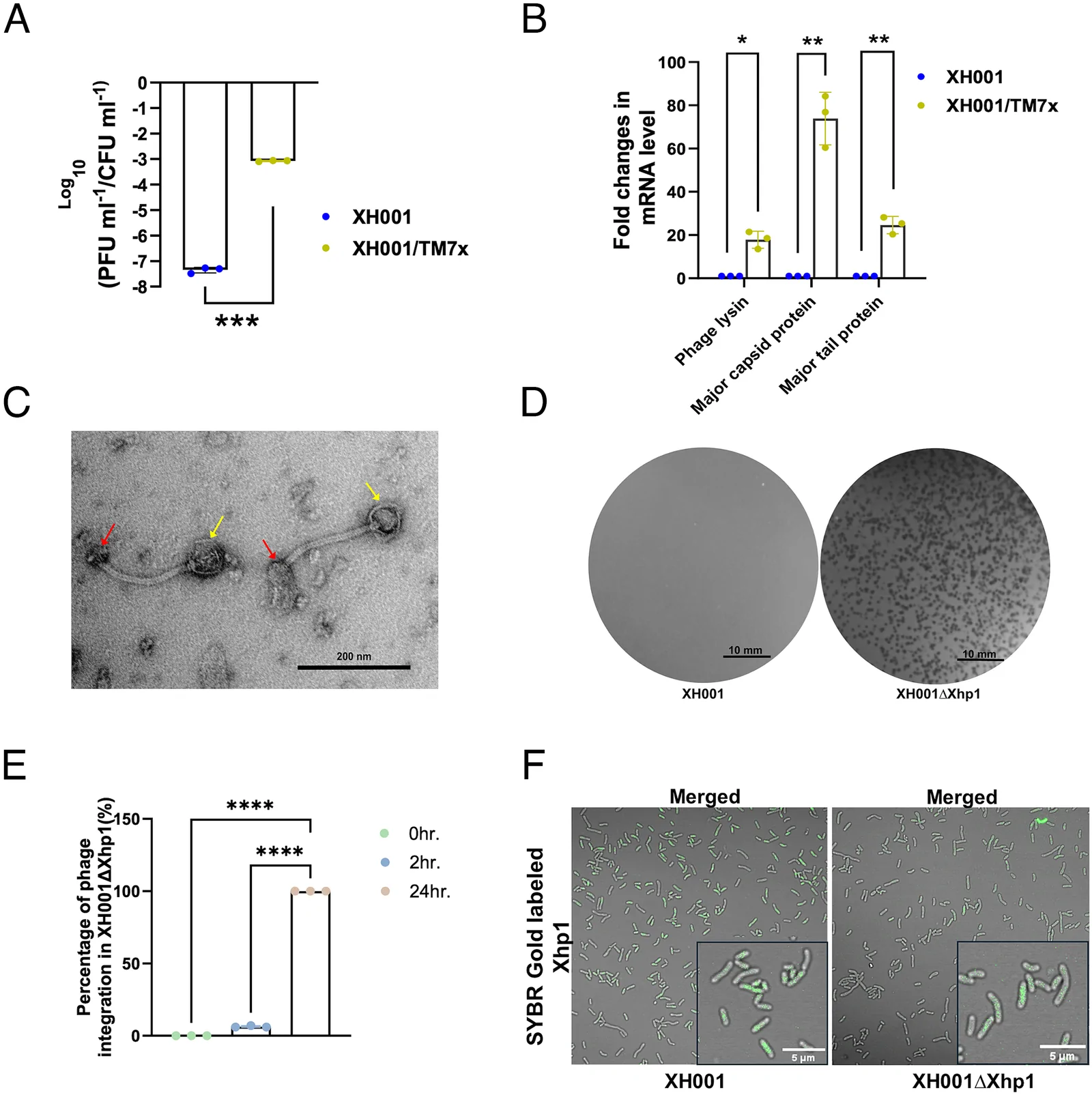
TM7x association enhances prophage induction in XH001. (*A*) Monoculture of XH001 and symbiotic coculture of XH001/TM7x were subjected to PFU (for Xhp1) and CFU (for XH001) analysis. PFU/CFU ratio was calculated to compare the activation of Xhp1 in XH001 vs. XH001/TM7x. The asterisks mark *P* < 0.001 (***) as calculated by Welch’s *t* test. (*B*) RT-PCR was performed to compare the expression of genes encoding phage lysin, major capsid protein, and major tail protein in XH001 and symbiotic coculture of XH001/TM7x. Data are presented as mean ± SE (n = 3). The statistical analysis was achieved by Welch’s *t* test: *P* < 0.01 (**), *P* < 0.05 (*). (*C*) Transmission electron microscopy (TEM) image showing representative images of purified phage particles isolated from XH001/TM7x coculture. Yellow and red arrows indicate the phage head and baseplate, respectively. (*D*) PFU assay was performed to determine the infectivity of Xhp1 against XH001 and XH001ΔXhp1. (Scale bar, 10 mm.) (*E*) The lysogenic conversion frequency was determined at 0, 2, and 24 h after isolated Xhp1 particles were added to XH001ΔXhp1 cells. The asterisks mark *P* < 0.0001 (****) as calculated by one-way ANOVA. (*F*) SYBR Gold-labeled Xhp1 particles were mixed with XH001 and XH001ΔXhp1, respectively. Samples were visualized under fluorescence and brightfield microscopy to monitor the binding between phage and bacterial cells. (Scale bar, 5 µm.)

Using XH001ΔXhp1, a XH001 derivative cured of Xhp1 (22), as a susceptible indicator strain in plaque-forming unit (PFU) assays, we found that coculture XH001/TM7x displayed a significantly higher PFU count than XH001 monoculture when normalized to the cell number of XH001 (Fig. 1*A*). Consistent with enhanced prophage induction, RT-PCR analysis revealed significantly elevated expression of multiple Xhp1 genes in the XH001/TM7x coculture, including those encoding the major capsid protein, major tail protein, and phage lysin (Fig. 1*B*).

To directly visualize released phage, Xhp1 particles were isolated from coculture supernatants and examined by TEM. The particles exhibited an icosahedral capsid with a long, noncontractile tail and a discernible baseplate-like structure, consistent with prior descriptions (22) of Xhp1 morphology (Fig. 1*C*). The abundance of Xhp1 particles recovered from the XH001/TM7x coculture strongly supports the conclusion that TM7x association promotes activation of the resident Xhp1 prophage.

### Xhp1 Displays Both Lytic and Lysogenic Infection Strategies Against XH001∆Xhp1.

Xhp1 efficiently lysed XH001ΔXhp1 on solid media, as indicated by clear plaques on agar plates (Fig. 1*D*). In contrast, exposure of planktonic XH001ΔXhp1 culture to Xhp1 did not result in a detectable decrease in bacterial CFU or an increase in PFU, even after extended incubation (*SI Appendix*, Fig. S1). This disparity suggested that growth state may influence Xhp1 infection outcomes.

To test whether planktonic conditions favor lysogenic conversion, we infected planktonic XH001ΔXhp1 with purified Xhp1 particles and monitored prophage integration over time using colony PCR targeting a prophage-encoded marker gene (*Materials and Methods*). Remarkably, prophage-carrying XH001 can already be detected 2 h. after the addition of Xhp1. More strikingly, after 24 h, all recovered colonies were positive for the marker gene (Fig. 1*E*), indicating complete lysogenic conversion. Together, these data demonstrate that Xhp1 can adopt distinct propagation strategies when encountering the same bacterial host under different growth modes: lytic infection on surface-grown and lysogenic conversion under planktonic conditions.

Consistent with prophage-mediated superinfection immunity, Xhp1 failed to infect wild-type XH001 harboring the resident Xhp1 prophage (Fig. 1*D*). This resistance was not attributable to impaired adsorption, as Xhp1 was able to bind efficiently to XH001 cells (Fig. 1*F*), suggesting that superinfection exclusion occurs at a postattachment stage (30, 31).

### Xhp1 Binds to the Episymbiont TM7x.

Due to its highly reduced genome and limited metabolic capacity, TM7x depends extensively on its host bacterium for growth and replication (6, 8). It has been proposed that episymbiotic CPR organisms may acquire host-derived cell wall components and retain surface-associated phage receptors (32). We therefore investigated whether Xhp1 particles could also bind to TM7x cells.

**Fig. 2.**
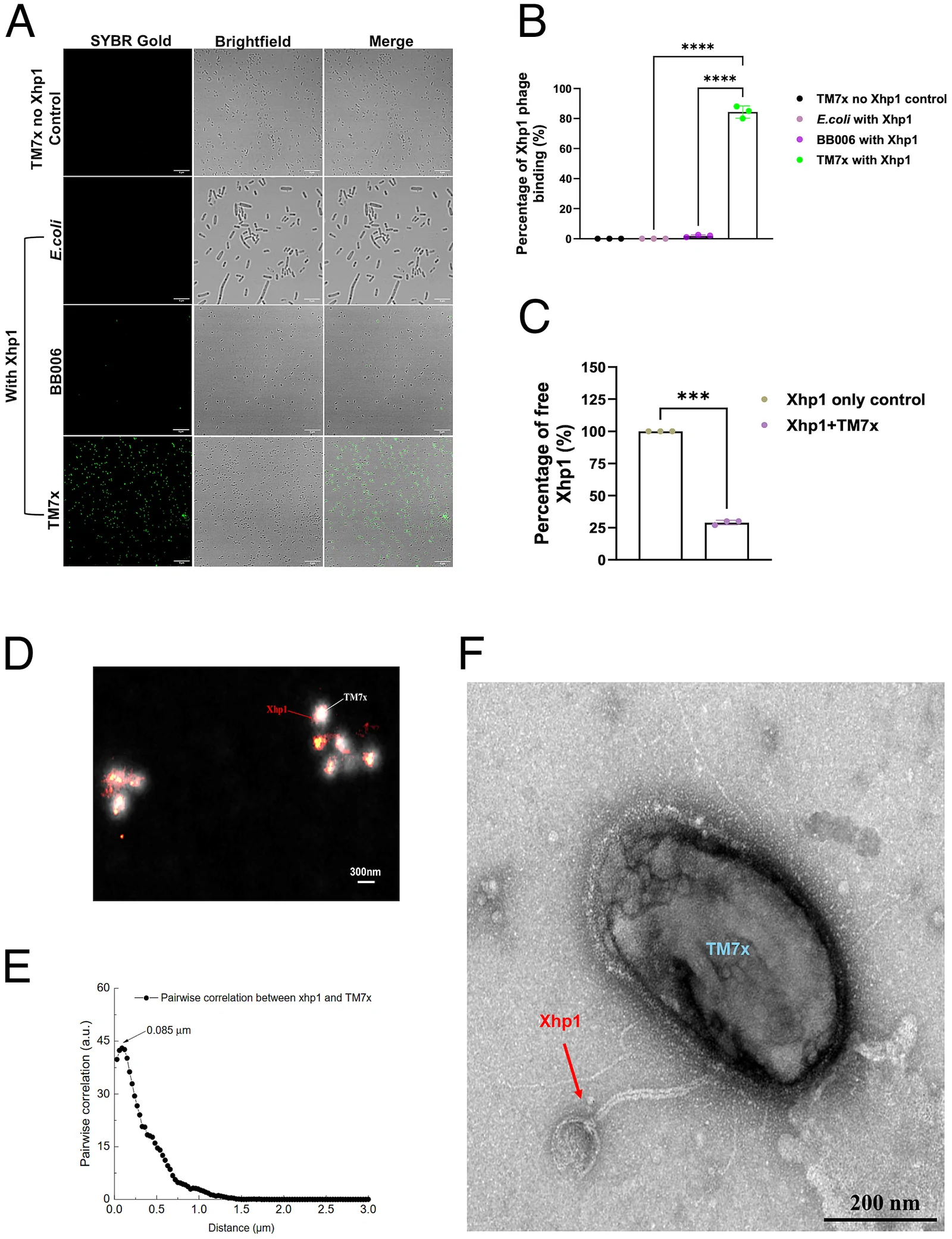
Xhp1 phage binds to TM7x. (*A*) SYBR Gold–labeled Xhp1 particles were mixed with either *E. coli*, BB006, or TM7x cells. Representative images were shown as the fluorescence channel, brightfield channel and merged fluorescence/brightfield channel. (Scale bar, 5 µm.) (*B*) Quantification of the percentage of bacterial cells bound by Xhp1 based on (*A*). The asterisks indicate a *P*-value of <0.0001, calculated by one-way ANOVA. (*C*) The adsorption assay to monitor the binding between Xhp1 and TM7x. The asterisks indicate a *P*-value of <0.0001(****), calculated by Welch’s *t* test. (*D*) Stimulated emission depletion (STED) microscopy reveals a close association of Xhp1 (red) with TM7x (white). (Scale bar, 300 nm.) (*E*) Pairwise correlation analysis of Xhp1 and TM7x signals shows strong short-range colocalization (≤0.085 µm) that decays to background levels at increasing dipole distances. (*F*) A TEM image shows the direct attachment of Xhp1 phage particles to the TM7x cell surface. (Scale bar, 200 nm.)

Using SYBR Gold-labeled Xhp1 in fluorescence-based binding assays (*Materials and Methods*), we observed strong colocalization of fluorescent signal with TM7x cells, indicating effective phage adsorption (Fig. 2*A*). In contrast, no appreciable binding was detected between Xhp1 and BB006 (33), another oral Saccharibacterial species that forms symbiosis with *Actinomyces* spp. F0309 but not XH001 (Fig. 2*A*). Meanwhile, no binding between Xhp1 and an *Escherichia coli* strain was observed (Fig. 2*A*). This is supported by quantitative analysis, which showed that while negligible binding could be observed between Xhp1 and *E. coli*, or BB006, close to 80% of the TM7x cells were bound by Xhp1 (Fig. 2*B*). Quantitative adsorption assay corroborated these findings, revealing a significant reduction (75%) in unbound Xhp1 particles following a 30 min incubation with TM7x (Fig. 2*C*).

We further employed high-resolution STED microscopy to confirm the physical interactions between phage and TM7x due to its excellent spatial resolution (34). This high-resolution imaging allows the visualization of both free Xhp1, as well as the Xhp1 particles binding to TM7x cells, as indicated by the colocalization of SPY650 signal (pseudo-red color for labeling Xhp1) and SPY595 signal [pseudo-white color for labeling TM7x (Fig. 2*D*)]. To have a quantitative analysis of the spatial proximity between Xhp1 and TM7x cells, we performed daime analysis (35). A pairwise correlation larger than 1 indicates a spatially aggregated behavior between two objects; a correlation value of 1 means random distribution; and a value smaller than 1 suggests repulsive interaction. Daime analysis showed a maximal pairwise correlation with a dipole distance of 85 nm, which was smaller than the typical size of TM7x cells (Fig. 2*E*). This further supports the binding between TM7x and Xhp1 particles.

Moreover, TEM provided the most direct visual evidence of Xhp1 adsorption to TM7x, with phage tail oriented perpendicular to the TM7x cell surface—an arrangement consistent with active attachment rather than nonspecific adherence (Fig. 2*F* and *SI Appendix*, Fig. S2*A*). TEM also showed multiple Xhp1 particles associated with individual TM7x cells and, in some cases, an apparent accumulation of phage particles on the TM7x surface (*SI Appendix*, Fig. S3).

Collectively, these data establish that Xhp1 particles released from XH001 can bind not only to their bacterial host but also to its episymbiont TM7x, revealing a previously unrecognized phage–symbiont interaction.

### Xhp1 Induced Limited Lytic Infection Against TM7x Cells.

**Fig. 3.**
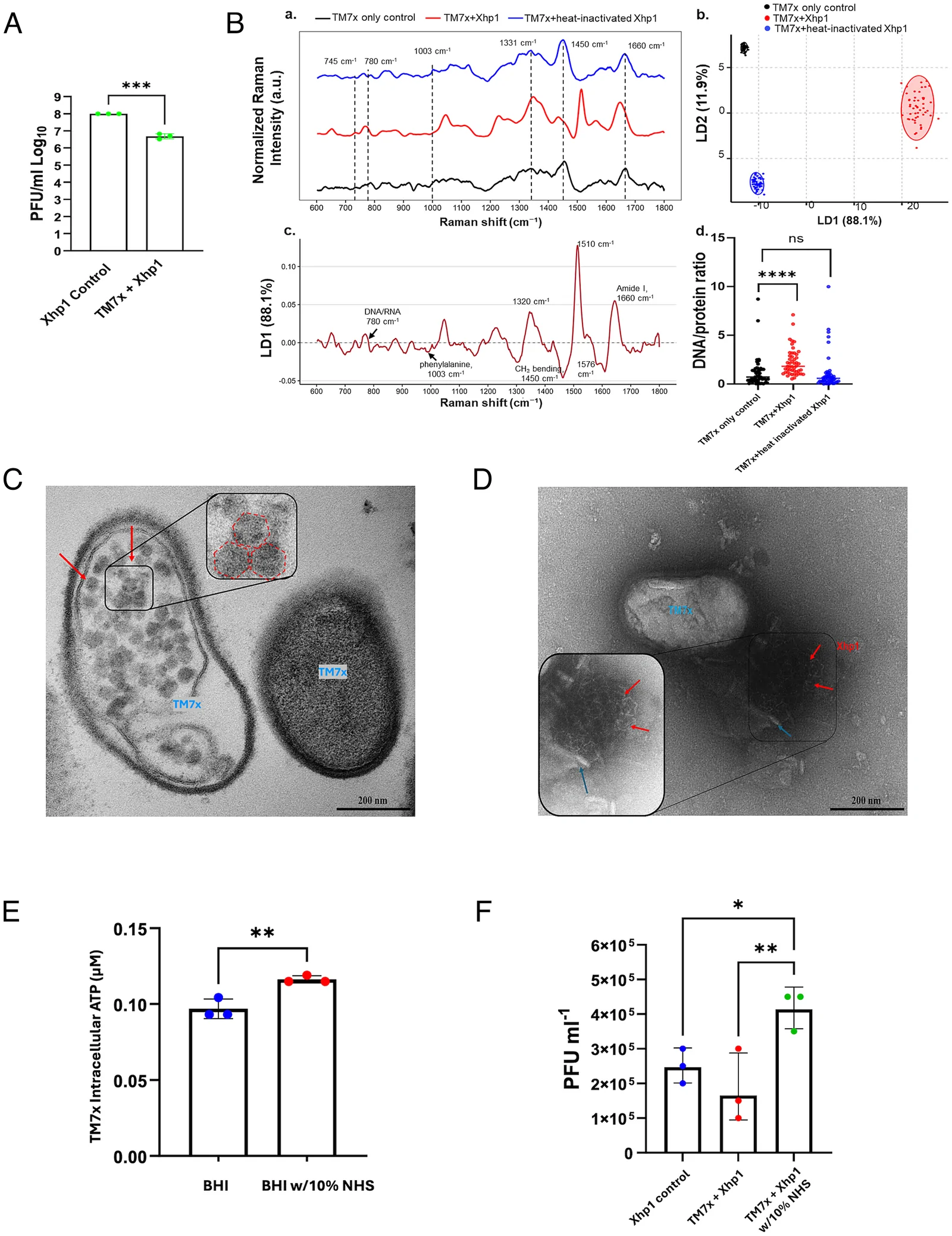
Xhp1 induces limited lytic infection in TM7x. (*A*) PFU assay was performed to determine the free Xhp1 particles 24 h after TM7x cells were infected with isolated Xhp1. Xhp1 only (without the addition of TM7x) was used as a control. The asterisks indicate a *P*-value of <0.001, calculated by Welch’s *t* test. (*B*) (*a*) Raman spectra of TM7x with Xhp1 or heat-killed Xhp1 (average of n = 50 Raman spectra per condition). Statistical analysis using PERMANOVA confirmed that the separation was significant (R^2^ = 0.33, *P* < 0.001). (*b*) LDA plot of Raman spectra demonstrating distinct clustering among three groups, indicating global metabolic shifts. (*c*) LD1 highlights Raman shifts that contribute most to the separation, showing condition-specific spectral features. (*d*) Quantitative analysis of integrated Raman intensities at key spectral markers: DNA (~780 cm^−1^) and protein (~1,250 and ~1,660 cm^−1^). Statistical comparisons were made using one-way ANOVA: *P* < 0.0001 (****), ns: not significant. (*C*) Representative thin-section TEM image demonstrating intracellular phage particles within TM7x cells. (Scale bar, 200 nm.) Red arrows indicate intracellular phage particles. (*D*) Representative TEM image showing TM7x cell lysis accompanied by the release of abundant phage particles. (Scale bar, 200 nm.) Red arrows indicate phage particles. (*E*) Intracellular ATP level of TM7x cells incubated in BHI vs. BHI supplemented with 10% heat-inactivated NHS. Statistical comparisons were made using one-way ANOVA: *P* < 0.01 (**). (*F*) PFU quantification of Xhp1 under different conditions. Statistical comparisons were made using one-way ANOVA: *P* <0.05 (*); *P* <0.01 (**).

Given the observed binding of Xhp1 to TM7x, we next investigated whether such interactions result in productive infection. When purified Xhp1 particles were added to isolated planktonic TM7x cells, there was an initial reduction in unbound Xhp1 due to the binding of phage particles to TM7x cells. Intriguingly, no increase in free phage particles was observed even after overnight incubation (Fig. 3*A*), suggesting that while Xhp1 particles bind to TM7x, they do not result in efficient population-level productive lytic infection under the conditions tested.

To assess physiological changes in TM7x induced by Xhp1 exposure, we used label-free Raman spectroscopy to examine intracellular biochemical alterations. Raman spectra were acquired from untreated TM7x, TM7x exposed to active Xhp1, and TM7x exposed to heat-inactivated Xhp1 (*Materials and Methods*). Analysis of the Raman fingerprint region (600 to 1,800 cm^–1^), which captures most intracellular biomolecular vibrations, revealed distinct spectral phenotypes among the three conditions (Fig. 3*B*). After vector normalization, characteristic peaks corresponding primarily to cytochrome c (745 cm^–1^), nucleic acids (780 cm^–1^), phenylalanine (1,003 cm^–1^), heme/porphyrin (1,510 cm^–1^), CH_2_ deformation (1,450 cm^–1^), and protein amide bands (1,250 and 1,660 cm^–1^) were detected. TM7x cells exposed to Xhp1 showed pronounced intensity changes at specific bands, including a marked increase at ~1,510 cm^–1^, relative to both control groups (Fig. 3 *B*, *a*).

To capture global spectral differences, we applied linear discriminant analysis (LDA). The two-dimensional LDA plot based on the first two linear discriminants demonstrated clear separation of Xhp1-exposed TM7x from untreated and heat-inactivated controls (Fig. 3 *B*, *b*). LD1 scores were significantly shifted in Xhp1-infected TM7x compared with both control groups (*P* < 0.001, unpaired Student’s *t* test), indicating a distinct biochemical state associated with phage exposure (Fig. 3 *B*, *b*).

Raman data also revealed a significant increase in intracellular nucleic acid–to–protein ratios, a signature of intracellular phage replication (36), in TM7x cells following Xhp1 exposure compared with both controls (Fig. 3 *B*, *c* and *d*). The finding suggests Xhp1 may achieve limited intracellular replication within certain TM7x cells, a possibility supported by TEM analysis of ultrathin-sectioned TM7x samples following phage exposure. A small subset, around 1% of TM7x cells analyzed (*SI Appendix*, Table S3), contained densely packed, hexagonally shaped intracellular particles resembling Xhp1 (Fig. 3*C*). In addition, a few, about 2% of total TM7x cells (*SI Appendix*, Table S3), were observed undergoing apparent lysis, releasing clusters of phage-like particles with icosahedral heads (Fig. 3*D* and *SI Appendix*, Fig. S2*B*). Furthermore, immunogold labeling TEM revealed the presence of the Xhp1-encoded glycerophosphodiester phosphodiesterase (GDPD_Xhp1_) within TM7x cells, providing independent evidence for active Xhp1 replication in its host (*SI Appendix*, Fig. S4). Phage-encoded GDPD is a baseplate-associated enzyme (37), a class of proteins that is incorporated into phage particles and functions extracellularly during adsorption and host cell-wall degradation to facilitate genome injection (38–40). As such, GDPD_Xhp1_ is not expected to be translocated into the host cytoplasm as part of the infection process. Therefore, detection of GDPD_Xhp1_ within TM7x cells is unlikely to represent passive delivery by infecting virions and instead indicates active phage gene expression. Given that synthesis of structural proteins is an essential step in virion assembly and productive phage propagation (39), these findings provide compelling evidence that Xhp1 replicates within TM7x cells.

The episymbiotic lifestyle of TM7x may explain the apparently limited productivity of infection at the population level. TM7x lacks many biosynthetic pathways and depends on XH001 for growth and metabolic support. Following physical separation from its host, TM7x remains viable but exhibits substantially reduced metabolic activity. Because bacteriophage replication depends heavily on host energy metabolism and biosynthetic capacity (41–43), a metabolically constrained TM7x cell may permit phage adsorption and limited intracellular replication while failing to efficiently complete the lytic cycle and release large numbers of progeny phage. To explore whether host physiological state influences Xhp1 replication, we performed additional experiments aimed at enhancing the metabolic activity of isolated TM7x cells. Previous work from our group demonstrated that, although metabolically limited, TM7x can directly acquire certain nutrients, including glucose and arginine, from the environment and use them to maintain viability in the absence of a host (19, 44). In addition, the increased abundance of TM7 in periodontal disease-associated communities suggests that host-derived factors present in serum-rich environments may support its physiology (16). We therefore investigated whether supplementation of BHI medium with 10% heat-inactivated normal human serum (NHS) could improve the metabolic activity of isolated TM7x cells and thereby increase their capacity to support Xhp1 replication.

Purified TM7x cells were incubated in BHI medium with or without 10% heat-inactivated NHS for 6 h, and TM7x cells were collected, and intracellular ATP levels were measured as an indicator of metabolic activity using an established method (19). TM7x cells cultured in NHS-supplemented medium exhibited significantly higher ATP levels than cells maintained in BHI alone (*P* < 0.01, Fig. 3*E*), indicating increased metabolic activity under this condition.

We next tested whether this enhanced metabolic state affected Xhp1 replication. Isolated TM7x cells were mixed with Xhp1 and embedded in soft-agar droplets containing either BHI alone or BHI supplemented with 10% heat-inactivated NHS. Following overnight incubation, released phages were quantified by plaque assay. Importantly, when infected with Xhp1, TM7x cultures supplemented with NHS produced significantly higher PFU counts than TM7x cultures maintained in BHI alone (*P* < 0.01, Fig. 3*F* and *SI Appendix*, Fig. S5). These data provide evidence that increased TM7x metabolic activity is associated with enhanced Xhp1 propagation and support the hypothesis that TM7x can serve as a biologically relevant host for Xhp1 replication.

### Phage Xhp1 Binding to TM7x Is Independent of Host Switching.

**Fig. 4.**
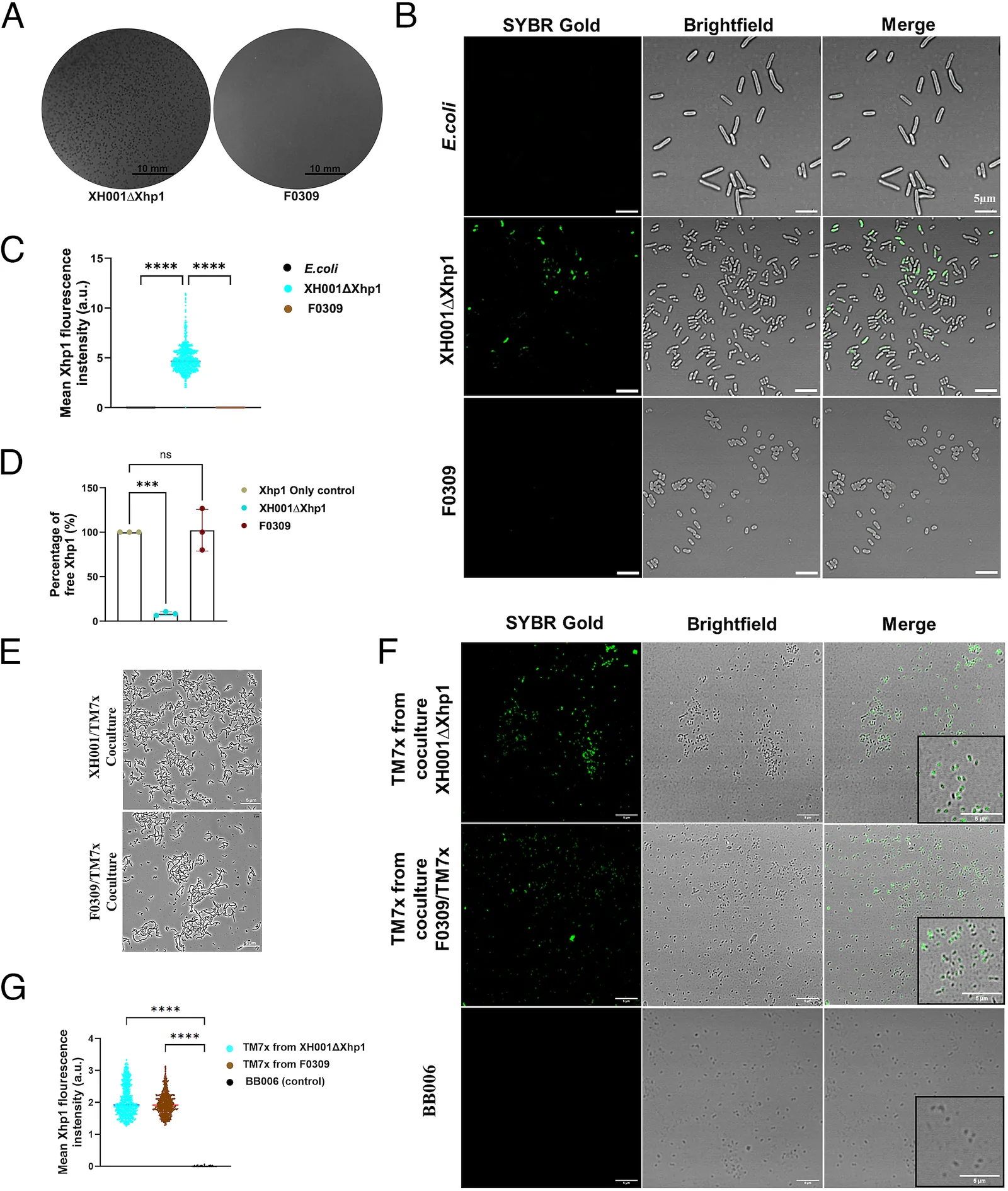
Phage Xhp1 binding to TM7x is independent of host switching. (*A*) Plaque assay was performed to determine the infectivity of Xhp1 against XH001ΔXhp1 and F0309. (Scale bar, 10 mm.) (*B*) SYBR Gold–labeled Xhp1 particles were mixed with either *E. coli*, XH001ΔXhp1, or F0309 cells. Representative images were shown as the fluorescence channel, brightfield channel, and merged fluorescence/brightfield channel. (Scale bar, 5 µm.) (*C*) Mean Xhp1 fluorescence intensity (a.u) was quantified for *E. coli*, XH001ΔXhp1, and *Actinomyces* spp. strain F0309. Statistical significance was assessed using one-way ANOVA: *P* < 0.0001 (****). (*D*) The adsorption assay to monitor the binding between XH001∆Xhp1 and F0309. Statistical significance was assessed using one-way ANOVA; *P* < 0.001(***). ns, not significant. (*E*) Representative images of the cocultures of XH001ΔXhp1/TM7x and F0309/TM7x. (Scale bar, 5 µm.) (*F*) SYBR Gold–labeled Xhp1 particles were mixed with either BB006, TM7x cells isolated from cocultures of XH001ΔXhp1/TM7x or F0309/TM7x. Representative images were shown as the fluorescence channel, brightfield channel, and merged fluorescence/brightfield channel. (Scale bar, 5 µm.) (*G*) Mean fluorescence intensity (a.u.) of SYBR Gold–labeled Xhp1 bound to TM7x from XH001ΔXhp1 or F0309, with BB006 as a negative control. Each point represents an individual cell. Statistical significance was assessed by one-way ANOVA: *P* < 0.0001 (****).

Given their highly reduced genome and limited metabolic capabilities, CPR organisms are predicted to depend extensively on their host bacterium for growth. It has therefore been proposed that episymbiotic CPR bacteria may incorporate host-derived cell wall components and thereby retain surface-associated phage receptors (32). To determine whether TM7x acquires Xhp1-binding receptors from its host bacterium, we examined phage attachment under different host–symbiont pairings. In addition to XH001, TM7x is known to form a stable episymbiotic association with *Actinomyces* spp. strain F0309 (45). We first demonstrated that in contrast to XH001∆Xhp1, F0309 is not susceptible to Xhp1 lytic infection, as evidenced by the absence of plaque formation in plaque assay (Fig. 4*A*).

We then assessed whether Xhp1 can bind directly to F0309. Using SYBR Gold–labeled phage particles, we observed a negligible fluorescence signal on F0309 cells by fluorescence microscopy (Fig. 4*B*), which was corroborated by quantitative analysis by measuring mean fluorescence intensity of fluorescently labeled phage associated with bacterial cells (Fig. 4*C*). Adsorption assays further showed that while incubation with XH001ΔXhp1 resulted in an approximately 90% reduction in unbound Xhp1 particles, no significant reduction in free Xhp1 particles was observed when phage was incubated with F0309 (Fig. 4*D*). These data indicate that F0309 does not support Xhp1 attachment and lacks functional Xhp1 receptors, consistent with its resistance to Xhp1 infection.

To test whether the bacterial host influences TM7x surface properties relevant to phage binding, we generated a F0309/TM7x coculture using an established protocol (7), in which TM7x relies on F0309 for growth and replication (Fig. 4*E*). Established coculture F0309/TM7x was passaged extensively to ensure TM7x in the coculture are newly replicated cells using F0309 as host. TM7x cells were isolated from cocultures of either F0309/TM7x or XH001ΔXhp1/TM7x and subjected to fluorescence-based Xhp1 binding assays. In both cases, SYBR Gold–labeled Xhp1 particles strongly colocalized with TM7x cells, whereas minimal signal was detected in the BB006 control (Fig. 4 *F* and *G*). Together, these results indicate that Xhp1 binding is an intrinsic property of TM7x and is independent of host switching.

### TM7x Reduces Lysogenic Conversion of XH001ΔXhp1 Under Planktonic Conditions.

We then proceeded to interrogate the impact of TM7x association on Xhp1-induced lysogenic conversion of XH001ΔXhp1 under planktonic conditions (46, 47). As cocultures of XH001ΔXhp1/TM7x are heterogeneous, comprising TM7x-attached and TM7x-free XH001ΔXhp1 cells as well as abundant free-floating TM7x cells generated through budding division (Fig. 4*E*). Given the demonstrated ability of TM7x to bind Xhp1, we hypothesized that free-floating TM7x could act as phage “sinks,” sequestering Xhp1 particles and thereby reducing lysogenic conversion of XH001ΔXhp1 in planktonic culture.

**Fig. 5.**
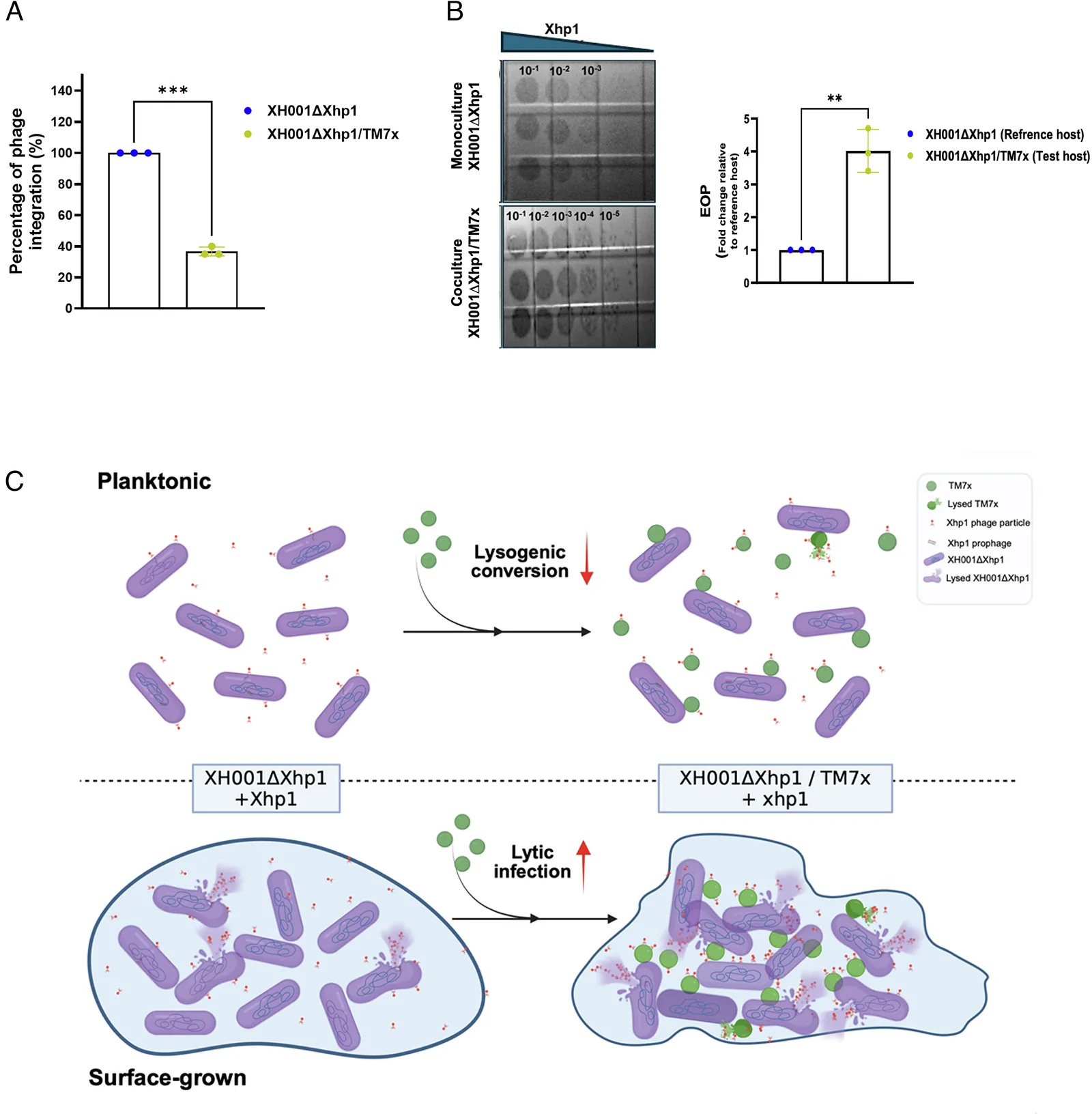
TM7x modulates Xhp1 integration and infection dynamics in a growth state–dependent manner. (*A*) The lysogenic conversion frequency was determined at 24 h after isolated Xhp1 particles were added to planktonic monoculture XH001ΔXhp1 or XH001ΔXhp1/TM7x symbiotic coculture. The asterisks indicate *P* < 0.001 (***), calculated by Welch’s *t* test. (*B*) EOP assay was performed to determine the sensitivity of XH001∆Xhp1 cells existing as a monoculture or episymbiotically associated with TM7x under surface-grown conditions. The asterisks indicate a *P* < 0.01 (**), calculated by Welch’s *t* test. (*C*) Graphical model illustrating the differential impact of the episymbiosis association of TM7x on the outcome of Xhp1 infection against XH001.

Consistent with this model, lysogenic conversion assays revealed that whereas XH001ΔXhp1 monocultures underwent complete lysogenic conversion within 24 h (Fig. 5*A*), fewer than 40% of XH001ΔXhp1 cells in episymbiotic coculture with TM7x became Xhp1-positive over the same period (Fig. 5*A*). These data suggest that TM7x lowers effective phage availability in planktonic environments by serving as phage “sink” and thereby mitigates Xhp1 infection of its host bacterium.

### TM7x Episymbiosis Enhances the Susceptibility of Surface-Grown XH001ΔXhp1 to Xhp1.

We next examined the impact of TM7x association on Xhp1 infection dynamics under surface-associated growth conditions, where Xhp1 induces productive lytic infection of XH001ΔXhp1 (Fig. 1*D*). Using efficiency-of-plating (EOP) assays, we found that XH001ΔXhp1 engaged in episymbiosis with TM7x exhibited a markedly higher EOP (of 4) compared with the same strain grown without TM7x (Fig. 5*B*). Thus, in contrast to planktonic conditions, TM7x association enhanced XH001ΔXhp1 susceptibility to Xhp1-mediated lysis on agar surfaces.

This apparent discrepancy can be reconciled by considering differences in spatial organization between growth conditions (Fig. 5*C*). In planktonic cultures, free-floating TM7x cells are often physically separated from XH001ΔXhp1, enabling them to act as distributed phage sinks that reduce the likelihood of Xhp1 encountering XH001ΔXhp1 cells. In contrast, surface growth constrains spatial separation, placing TM7x—including newly detached daughter cells—in close proximity to XH001ΔXhp1. Under these conditions, Xhp1 binding to TM7x, as well as Xhp1-induced lytic infection of a small fraction of the subpopulation (Fig. 3 *C* and *D*), may locally concentrate phage particles near XH001ΔXhp1 cells, thereby increasing effective phage dosage and enhancing lytic infection.

## Discussion

CPR bacteria comprise a major fraction of bacterial diversity, yet their interactions with phages have remained largely untested experimentally (3, 5). Although genomic surveys have predicted widespread phage exposure among CPR lineages, the absence of cultivated systems has prevented direct validation. Using the experimentally tractable CPR model currently available, *N. lyticus* TM7x, we show that a temperate phage encoded by its bacterial host can bind to, infect, and replicate within TM7x. While infection efficiency is limited under the conditions tested, our findings provide direct experimental support for the inclusion of CPR bacteria within phage interaction networks and reveal phages as context-dependent modulators of CPR–host symbioses.

### Episymbiosis Promotes Prophage Induction in a Structured Microbial Niche.

We demonstrate that the episymbiotic association of TM7x with XH001 enhances the induction of prophage Xhp1. TM7x association is known to impose physiological and regulatory stress on XH001 (8), and cellular stress is a well-established trigger for prophage activation (29). Thus, rather than representing stochastic induction, Xhp1 activation appears to be a predictable outcome of symbiotic interaction. In the oral microbiome, where dense multispecies biofilms dominate and physical associations are extensive (48), such stress-responsive prophage induction may have disproportionate effects. *Actinomyces/Schaalia* species are abundant early colonizers of dental plaque (16, 49, 50), and Saccharibacteria (TM7) lineages are prevalent members of both healthy and dysbiotic oral communities (16, 51, 52). Prophage induction within these taxa could therefore influence local viral loads, alter competitive dynamics, and indirectly affect community assembly within biofilm microenvironments.

### Environmental Context Shapes Phage Infection Strategies.

Xhp1 adopts distinct infection strategies depending on host growth state, producing lytic infection on surfaces while favoring lysogenic conversion under planktonic conditions (Figs. 1*D* and 5*A*). This flexibility is consistent with temperate phage strategies that balance short-term replication with long-term persistence across fluctuating environments (27, 53). Surface-associated growth likely approximates biofilm conditions in the oral cavity, where spatial confinement and high cell densities can favor lytic propagation, whereas planktonic states may select for lysogeny. Importantly, these outcomes are further shaped by the presence of TM7x, underscoring the importance of community context in phage biology. Our results indicate that phage–host dynamics inferred from monocultures may not accurately reflect infection outcomes in multispecies assemblages characteristic of natural microbiomes.

### Direct Phage Interaction with a CPR Bacterium.

A key finding of this study is that Xhp1 particles bind specifically to TM7x cells and can undergo limited intracellular replication under the conditions tested. Evidence from fluorescence-based binding assays, superresolution fluorescence microscopy, TEM, and biochemical signatures collectively supports a genuine infection process rather than nonspecific adsorption. Although productive lysis of TM7x occurs only in a small subset of cells, intracellular phage particles and altered cellular Raman signatures consistent with phage replication indicate that TM7x can function as a phage host under certain conditions. Rather than contradicting evolutionary expectations, the limited efficiency of infection may reflect constraints imposed by CPR biology. Streamlined genomes, reduced metabolic capacity, and obligate dependence on host-associated growth may restrict phage replication, leading to low-frequency or condition-dependent infections. Nevertheless, even rare infection events could impose sufficient selective pressure to maintain antiviral defense systems encoded in some CPR genomes.

### Host-Independent Binding Highlights Conserved CPR Surface Features.

Our host-switching experiments demonstrate that TM7x binding to Xhp1 is independent of the specific bacterial partner, indicating that TM7x does not acquire phage receptors from its host. Rather, surface features intrinsic to TM7x are likely responsible for mediating phage attachment. This finding supports the idea that, despite their extensive metabolic dependence on host bacteria, members of the CPR maintain conserved cell envelope architectures across different host contexts.

At present, the specific receptors in TM7x and XH001 that mediate Xhp1 binding remain unidentified, and the molecular basis for host-dependent modulation of receptor expression is unclear. Future studies aimed at defining the molecular determinants governing Xhp1–TM7x interactions across different host backgrounds will be essential for understanding how phage pressure shapes CPR cell envelope evolution within multispecies microbial communities.

### TM7x Modulates Phage Availability in a Spatially Dependent Manner.

TM7x exerts opposing effects on Xhp1 infection of XH001 depending on spatial organization. Under planktonic conditions, free-floating TM7x cells reduce lysogenic conversion of XH001ΔXhp1, consistent with TM7x acting as a phage sink that lowers effective phage availability. In contrast, during surface-associated growth, TM7x increases the susceptibility of XH001ΔXhp1 to lytic infection, likely by locally concentrating phage particles within a constrained niche. These findings suggest that CPR bacteria can indirectly regulate viral encounter rates within microbial communities. In spatially structured environments such as oral biofilms, where diffusion is limited and microscale organization dominates, such effects may influence infection probabilities and population-level outcomes.

Together, our results support an eco-evolutionary framework in which phages act as mediators of multilevel (54–56) selection in CPR-containing systems. Prophage induction links bacterial host physiological state to viral propagation, while phage interactions with CPR symbionts generate feedback that can either buffer or amplify infection pressure on bacterial hosts, depending on environmental context. Rather than acting primarily as agents of CPR mortality, phages may modulate the stability and evolutionary trajectories of CPR–host associations. Although derived from a single model system, this study provides a conceptual foundation for integrating CPR bacteria into phage ecology.

### Limitations and Future Directions.

This study is limited to a single, experimentally tractable CPR–host system and a single temperate phage, and therefore, the extent to which our findings generalize across the phylogenetically diverse CPR remains uncertain. Infection of TM7x by Xhp1 occurs at low frequency under laboratory conditions. While our data showed that Xhp1 can infect TM7x and undergo intracellular replication, they do not demonstrate robust productive infection or extensive progeny amplification across the TM7x population. Thus, it remains unclear whether such infections are more prevalent under specific physiological states, environmental cues, or community contexts encountered in vivo. Meanwhile, the proposed phage sink model and TM7x-mediated local phage-concentration effect are consistent with, but not directly demonstrated by, our data. Future work is required to determine whether TM7x significantly influences Xhp1 availability, dissemination, or infection outcomes in mixed microbial communities and to establish the ecological relevance of these proposed mechanisms. Future work leveraging additional cultivated CPR models, in situ approaches, and multispecies biofilm systems will be essential to determine how widespread CPR–phage interactions are, to identify the molecular determinants of phage adsorption and susceptibility in CPR bacteria, and to establish how phage-mediated processes influence CPR persistence, host bacteria fitness, and community structure over ecological and evolutionary timescales.

## Materials and Methods

### Bacterial Strains and Growth Conditions.

*S. odontolytica* strain XH001 and the XH001/TM7x coculture were isolated from the human oral cavity under University of California, Los Angeles, Institutional Review Board No. 09-08-068-02A. Informed consent was obtained from participants prior to sample collection in accordance with the approved IRB protocol. The bacterial cultures were maintained as frozen stocks as previously described (6). The prophage-cured derivative XH001ΔXhp1 and the corresponding coculture TM7x/XH001ΔXhp1 were generated and maintained as frozen stocks as reported previously (22). All bacterial strains and phages used in this study are listed in *SI Appendix*, Table S1. XH001, XH001ΔXhp1, XH001/TM7x, XH001ΔXhp1/TM7x, F0309, and F0309/TM7x were cultured in brain heart infusion (BHI; Thermo Fisher Scientific, MA) at 37 °C under microaerophilic (2% O_2_, 5% CO_2_, balanced with N_2_) condition.

### Isolation of Free-Floating TM7x Cells.

TM7x cells were isolated from cocultures using previously described protocols to minimize host bacterial debris contamination (7, 8, 20, 44). Briefly, ~400 mL of XH001∆Xhp1/TM7x or F0309/TM7x coculture grown in BHI was transferred into 50 mL tubes, vortexed to dislodge loosely attached TM7x, and centrifuged at 3,000×*g* for 5 min to pellet host cells. Supernatants were filtered through 0.45-μm PVDF filters (Millipore Sigma) to remove residual host bacteria, followed by centrifugation at 20,000×*g* for 1 h at 4 °C to pellet TM7x cells. Phase-contrast microscopy (Nikon Eclipse E400) was used to confirm the presence and morphology of isolated TM7x cells. For storage, purified TM7x preparations were adjusted to an OD_600_ of 0.2 to 0.4 and stored at −80 °C in phosphate-buffered saline (PBS) supplemented with 15% glycerol.

### TM7x Host Attachment Assay.

Episymbiotic cocultures were established as previously described (7, 8). Briefly, isolated TM7x cells were normalized to an OD600 of 0.1 and added to XH001∆Xhp1 or F0309 at a multiplicity of infection (MOI) of 10. Cocultures were incubated at 37 °C under microaerophilic condition and passed every 24 h for up to 15 passages. Following the establishment of stable cocultures, TM7x cells were isolated using the protocol described above.

### Raman Spectroscopy Analysis.

Raman spectroscopy analysis was performed as previously described (36, 44, 57, 58). TM7x only or TM7x mixed with Xhp1 were incubated for 2 h at 37 °C. TM7x cells mixed with heat-inactivated Xhp1 (75 °C, 30 min) were used as a control. Cells were then fixed in 10% neutral-buffered formalin (Sigma-Aldrich) for 60 min at room temperature, washed three times with sterile water, and air-dried on aluminum-coated Raman substrates. Raman spectra were collected using an HR Evolution confocal Raman microscope (Horiba Jobin-Yvon, France) equipped with a 532 nm Nd:YAG laser. The laser output was attenuated to 8 mW at the sample using 25% neutral density filters. A 100× air objective (NA = 0.6) was employed to focus the laser onto TM7x cell aggregates, producing a spot size of approximately 1 μm^2^. TM7x cells tend to form small aggregates, so the laser may occasionally capture signals from multiple cells, contributing to spectral variability. Scattered Raman signals were detected using a charge-coupled device (CCD) detector cooled to −70 °C. Spectra were recorded over the ranges 600 to 1,800 cm^−1^, using a 600-groove/mm diffraction grating, providing a spectral resolution of 1.5 cm^−1^. Acquisition settings included a 3-s integration time and two accumulations per spectrum. Spectra were processed in LabSpec 6 (cosmic ray removal, baseline correction, vector normalization) and analyzed in RStudio (2024.09.1)

### Phage Induction, Isolation, and Purification.

Prophages were induced from mid-log-phase cultures of *S. odontolytica* (OD_600_ ≈ 0.5) using mitomycin C (0.1 µg mL^–1^) and incubated for 6 h. Cell debris was removed by centrifugation (8,000×*g*, 10 min), followed by filtration through 0.22 µm membranes. Phage particles were precipitated by the addition of NaCl (final concentration 1 M) and polyethylene glycol 8000 (10% wt/vol) and incubated overnight at 4 °C. Precipitates were collected by centrifugation (10,000×*g*, 30 min), resuspended in SM buffer [50 mM Tris-HCl (pH 7.5), 100 mM NaCl, 8 mM MgSO_4_] and further purified by CsCl density-gradient ultracentrifugation (100,000×*g*, 2 h, 4 °C). Visible phage bands were collected, dialyzed against SM buffer, and stored at 4 °C (59).

### Phage Infection within Soft Agar.

Isolated TM7x cells were mixed with Xhp1 and embedded in 0.2% soft-agar droplets containing either BHI alone or BHI supplemented with 10% heat-inactivated NHS. Following overnight incubation, agar droplets were collected, disrupted, and vortexed in 1 mL of BHI, and incubated overnight to release phages, which were further quantified by PFU assay.

### PFU Assay.

Phage titers were determined using the double-layer agar method (60). Tenfold serial dilutions of phage lysates were prepared in SM buffer and mixed with host cells (XH001ΔXhp1), then incubated for 10 min. The mixtures were combined with molten 0.3% soft agarose and overlaid onto BHI agar plates. After incubation for 18 to 48 h, plaques were enumerated, and titers were expressed as PFU mL^–1^.

### Phage Adsorption Assay.

Adsorption assays were performed by mixing phage Xhp1 with mid-log-phase host cells (XH001ΔXhp1 or TM7x/XH001ΔXhp1) at a MOI of 100 and incubating for 15 min at 37 °C. Samples were centrifuged (7,000×*g*, 5 min), and unadsorbed phages in the supernatant were quantified by plaque assay. Adsorption efficiency was calculated as the percentage reduction in free phage relative to the initial input (61).

### EOP Assay.

The EOP was determined to assess phage infectivity against XH001ΔXhp1 and XH001ΔXhp1/TM7x. Serial dilutions of phage lysates were spotted onto bacterial lawns using the double-layer agar method. Plates were incubated until plaques were visible, and PFU counts obtained on test strains were normalized to those on the reference host to calculate EOP values (62).

### TEM.

To examine phage particles and TM7x–host interactions, cocultures with or without phage exposure were fixed in 2.5% glutaraldehyde in PBS. For thin-section TEM, pelleted cells were fixed for at least 2 h at room temperature in fixative containing 2.5% glutaraldehyde, 1.25% paraformaldehyde, and 0.03% picric acid in 0.1 M sodium cacodylate buffer (pH 7.4). Samples were washed in cacodylate buffer and postfixed for 1 h in 1% osmium tetroxide containing 1.5% potassium ferrocyanide. After washing with water and maleate buffer, samples were stained with 1% uranyl acetate for 1 h, dehydrated through graded ethanol series, and transferred to propylene oxide. Samples were infiltrated overnight in a 1:1 mixture of propylene oxide and Spurr’s low-viscosity resin (Electron Microscopy Sciences, Hatfield, PA), embedded in resin, and polymerized at 60 °C for 48 h. Ultrathin sections (~60 nm) were cut using a Reichert Ultracut-S microtome, mounted on copper grids, stained with lead citrate, and examined using a transmission electron microscope (6).

### Immunogold TEM.

The Xhp1-encoded glycerophosphodiester phosphodiesterase (GDPD_Xhp1_), a predicted baseplate-associated protein, was cloned, expressed, and purified as described in *SI Appendix*, *Supplemental Materials and Methods*. The purified recombinant GDPD_Xhp1_ protein was used to generate a rabbit polyclonal antibody through Pacific Immunology Corp. (Pacific Immunology, CA). To assess Xhp1 replication in TM7x cells, TEM was performed on Xhp1-infected TM7x samples using the anti-GDPD_Xhp1_ antibody. Ultrathin sections were incubated with the rabbit polyclonal anti-GDPD_Xhp1_ primary antibody, followed by a 10 nm gold-conjugated goat anti-rabbit IgG secondary antibody. After staining, samples were examined by TEM.

### Real-Time qRT-PCR.

Bacterial cultures were grown overnight in BHI liquid medium or on BHI agar plates at 37 °C under microaerophilic conditions. Cells were collected and resuspended in TRIzol reagent (Invitrogen, Carlsbad, CA) for total RNA extraction. RNA was reverse transcribed using the PrimeScript™ RT Reagent Kit (Takara, Japan). qRT–PCR was performed on a CFX96 real-time PCR system (Bio-Rad, USA) using the Powerup SYBR green master mix (Applied Biosystems). The 16S rRNA gene served as the internal reference, and relative gene expression was calculated using the 2^–ΔΔCt^ method. Primers used in this study are listed in *SI Appendix*, Table S2.

### Fluorescence Microscopy.

Purified Xhp1 phage particles (approximately 10^9^ to 10^10^ PFU mL^–1^) were fluorescently labeled with SYBR Gold (1:10,000 dilution) by overnight incubation in the dark. Excess dye was removed by centrifugal filtration. Bacterial cultures at mid-log phase were washed with PBS and incubated with labeled phage for 30 to 60 min at room temperature. Samples were washed to remove unbound phages, fixed with 4% paraformaldehyde, mounted on glass slides, and images were acquired by a confocal laser scanning microscope (ZEISS LSM880) equipped with a 63× oil immersion objective with an excitation wavelength of 488 nm and an emission collection window from 500 to 550 nm (63).

### STED Microscopy.

STED microscopy was performed to achieve superresolution imaging of phage–TM7x interactions. Samples were prepared as described for fluorescence microscopy and imaged using a STED microscope (STEDYCON). Phage particles were labeled by a DNA stain, SPY650. And TM7x cells were labeled by another DNA stain, SPY595. The depletion laser used in this experiment is 775 nm, and the two fluorophores were excited by 640 nm and 561 nm, respectively. From the same field of view acquired under a 100× oil objective (NA = 1.45), both the normal confocal fluorescence images and STED images were obtained. Exposure time and laser intensity were optimized to harvest the superresolution images with the finest resolution (~50 nm) and minimal photobleaching. Analysis of the obtained images was conducted by FiJi (64, 65).

### Lysogenic Conversion Experiment.

Lysogenic conversion of XH001ΔXhp1 cells, either within monoculture or XH001ΔXhp1/TM7x coculture, to XH001 was performed by challenging the planktonic XH001ΔXhp1 monoculture or XH001ΔXhp1/TM7x coculture with phage Xhp1. Overnight cultures of XH001ΔXhp1 or XH001ΔXhp1/TM7x were diluted to an optical density at 600 nm (OD_600_) of 0.2. A defined volume of the bacterial culture was mixed with Xhp1 phage at an MOI of 10:1 (Xhp1: XH001). The mixture was incubated under appropriate growth conditions, and samples were collected at 0 (immediately after mixing), 6, and 24 h after mixing. At each time point, aliquots were collected for the determination of lysogenic conversion. Briefly, samples were serially diluted, plated on BHI agar plates, and incubated at 37 °C in microaerophilic conditions for colonies to grow. From each replicate, 30 colonies were randomly selected and screened for the integration of Xhp1 using Xhp1-specific integrase primers (*SI Appendix*, Table S2) by PCR under the following conditions: initial denaturation at 94 °C for 4 min; followed by amplification cycles of 94 °C for 30 s, 57 °C for 30 s, and 72 °C for 30 s; with a final extension at 72 °C for 5 min. Reaction mixtures were run on agarose gel and stained for PCR product visualization.

### ATP Quantification.

Purified TM7x cells were incubated in BHI medium supplemented with or without 10% heat-inactivated NHS for 6 h under microaerobic conditions. Following incubation, TM7x cells were harvested and intracellular ATP levels were quantified using the ATP Colorimetric/Fluorometric Assay Kit (Abcam, Cat. No. ab83355/K354) according to the manufacturer’s instructions. ATP levels were calculated from a standard curve generated using ATP standards supplied with the kit.

### Statistical Analysis.

All statistical analyses were performed using Prism v10.3.1 (GraphPad Software). Data are presented as mean ± SE (n = 3). Depending on the experimental design, unpaired Student’s or Welch’s tests or one-way ANOVA were applied. Statistical significance was defined as *P* < 0.05 (*), *P* < 0.01 (**), *P* < 0.001 (***), or *P* < 0.0001 (****).

## Supplementary Material

Appendix 01 (PDF)

## Data Availability

The Raman data are accessible at https://github.com/nusratnahar17/Prophage-project/blob/main/R script (66). All study data are included in the article and/or supporting information.
