# Supplementary material for: A host-encoded prophage targets a Candidate Phyla Radiation bacterium and shapes episymbiotic interactions
Source: Proc Natl Acad Sci U S A. 2026 Sep 8;123(37):e2615890123. doi: 10.1073/pnas.2615890123 (PMC13578794; doi:10.1073/pnas.2615890123)
Supplement: Supplementary file 1 [file pnas.2615890123.sapp.pdf]

PNAS
www.pnas.org

Supplementary Information for

A Host-Encoded Prophage Targets a Candidate Phyla Radiation Bacterium and Shapes Episymbiotic Interactions

Ajay Kumar, Nusrat Nahar, Deepak Chouhan, Jeffrey S. McLean, Batbileg Bor, Pu-Ting Dong*, and Xuesong He*

* Corresponding author:
Pu-Ting Dong, Email: puting.dong@stonybrook.edu
Xuesong He, Email: xhe@forsyth.org

This PDF file includes:

Supplementary Materials and Methods
Figures S1, S2, S3, S4 and S5.
Tables S1, S2, S3 and S4.

Supplementary Materials and Methods

Expression and purification of GDPD$_{Xhp1}$ protein

The full-length *gdpd*$_{Xhp1}$ coding sequence was cloned (Table S4) into the pET28a expression vector to generate an N-terminal His-tagged fusion protein. The construct was sequence-verified and transformed into *E. coli* BL21(DE3) for protein overexpression. Cultures were grown under standard induction conditions with 0.5 mM IPTG, harvested by centrifugation at 5,000 × g for 10 min at 4 °C, and cell pellets were resuspended in lysis buffer consisting of 50 mM NaH_2PO_4, 300 mM NaCl, and 20 mM imidazole (pH 8.0). Cells were lysed by sonication on ice at 50% amplitude using 10-s pulses for a total sonication time of 60 s per sample. Lysates were clarified by centrifugation at 12,000 × g for 10 min at 4 °C, and the soluble fraction was retained for purification. His-tagged GDPD$_{Xhp1}$ was purified by immobilized metal affinity chromatography (IMAC) using an ÄKTA fast protein liquid chromatography (FPLC) system pre-equilibrated with IMAC binding buffer (50 mM NaH_2PO_4, 300 mM NaCl, 20 mM imidazole, pH 8.0). Clarified lysates were loaded onto a Ni^{2+}-charged affinity column, washed extensively with binding buffer to remove nonspecifically bound proteins, and eluted with buffer containing 300 mM imidazole. Elution fractions were monitored by absorbance at 280 nm, collected, and analyzed by SDS-PAGE. Fractions containing purified GDPD$_{Xhp1}$ were pooled, dialyzed, and concentrated using a 10-kDa molecular-weight-cutoff centrifugal concentrator (Sigma-Aldrich, Saint Louis, MO).

Supplementary Figures:

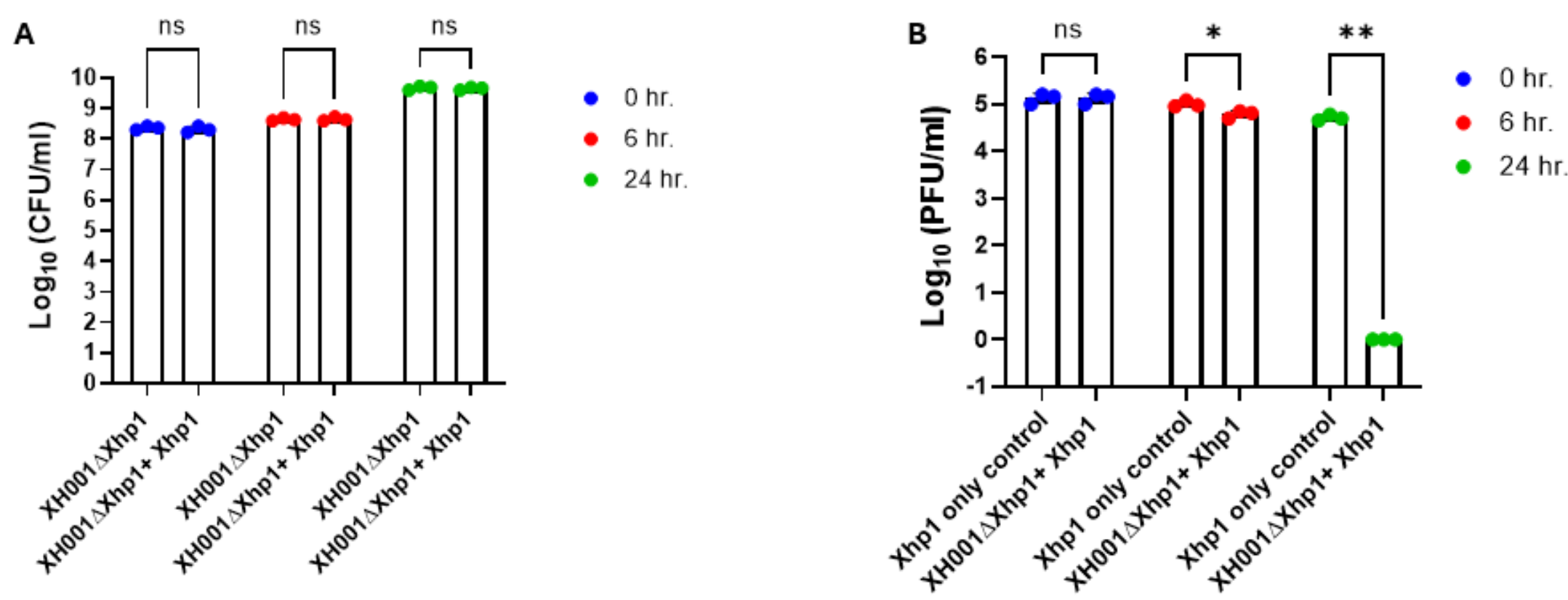

Fig. S1. In planktonic cultures, phage Xhp1 does not reduce CFU of XH001ΔXhp1 or increase PFU of Xhp1. (A) Longitudinal monitoring of XH001ΔXhp1 CFU post-challenge with or without phage Xhp1; **(B)** Longitudinal monitoring of Xhp1 PFU after challenging XH001ΔXhp1 with phage Xhp1.

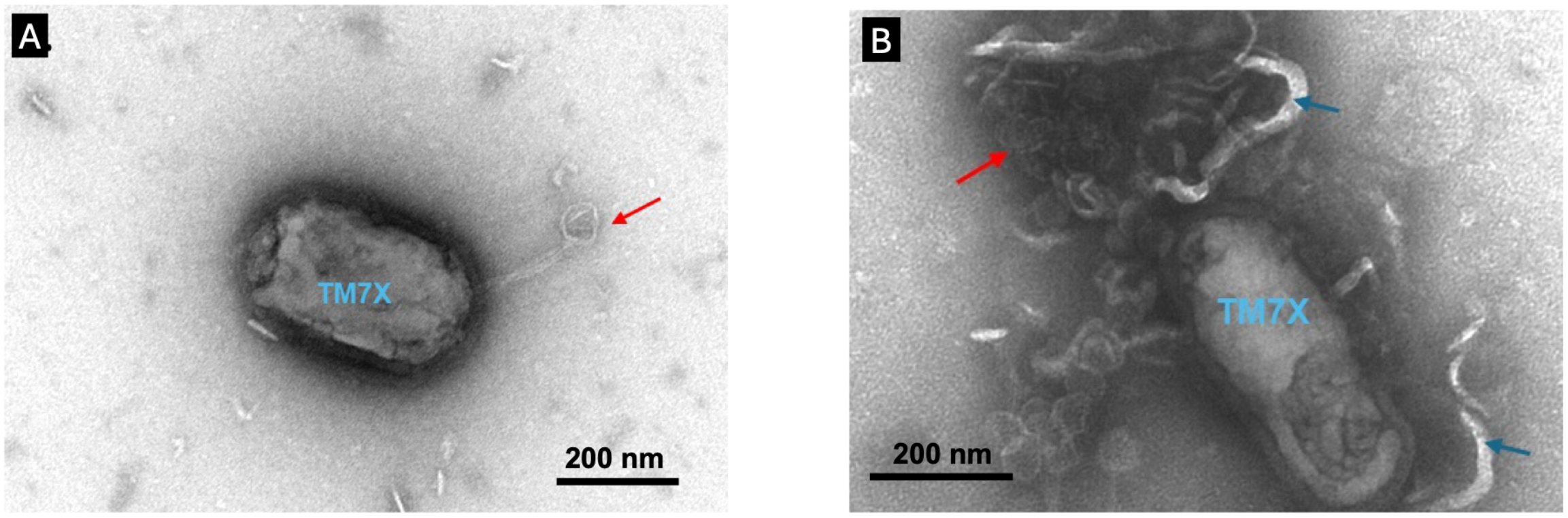

Fig. S2: Transmission electron microscopy (TEM) images showing (A) direct attachment of Xhp1 phage particles to the TM7x cell surface. Red arrows indicate bound phage particle; (B) Lysis of Xhp1-infected TM7x cell. Red arrows indicate phage particles; Blue arrows indicate lysed TM7x debris. Scale bar, 200 nm.

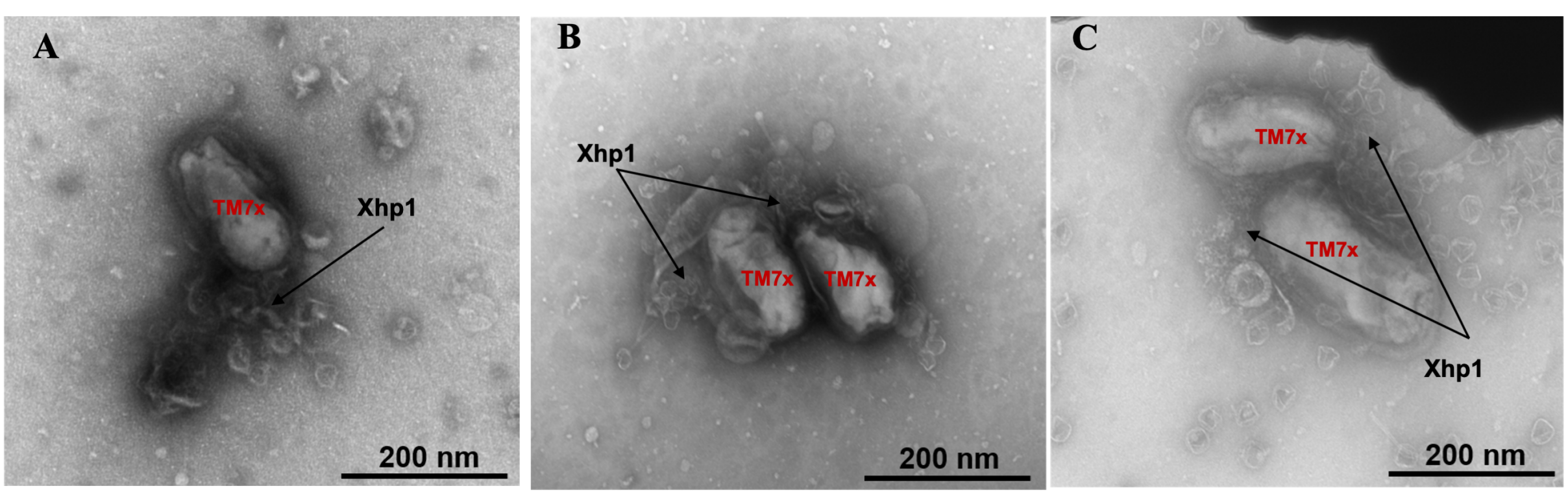

Fig. S3: Transmission electron microscopy (TEM) images showing multiple Xhp1 particles associated with individual TM7x cells (A-C). Scale bar, 200 nm.

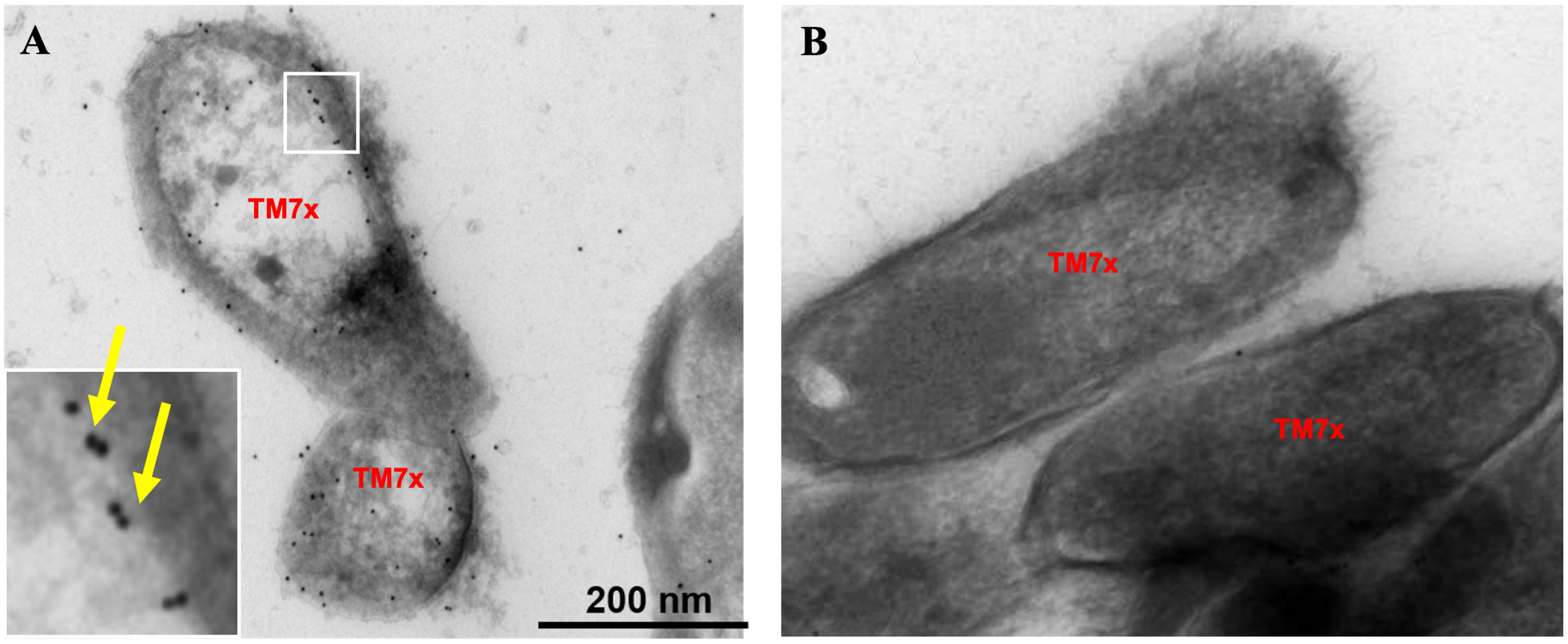

Fig. S4: Transmission electron micrographs of immunogold-labeled GDPD$_{xhp1}$ protein. Immunogold TEM was performed using a rabbit polyclonal antibody raised against the purified Xhp1-encoded glycerophosphodiesterase (GDPD$_{xhp1}$) protein. Ultrathin sections of Xhp1-infected TM7x samples were incubated with the anti-GDPD primary antibody, followed by a 10 nm gold-conjugated goat anti-rabbit IgG secondary antibody. After staining, samples were examined by TEM, and gold particle labeling was used to localize the GDPD$_{xhp1}$ protein within TM7x cells. **(A)** TM7x infected with Xhp1. Yellow arrows indicate gold particles. **(B)** TM7x without Xhp1 as a control.

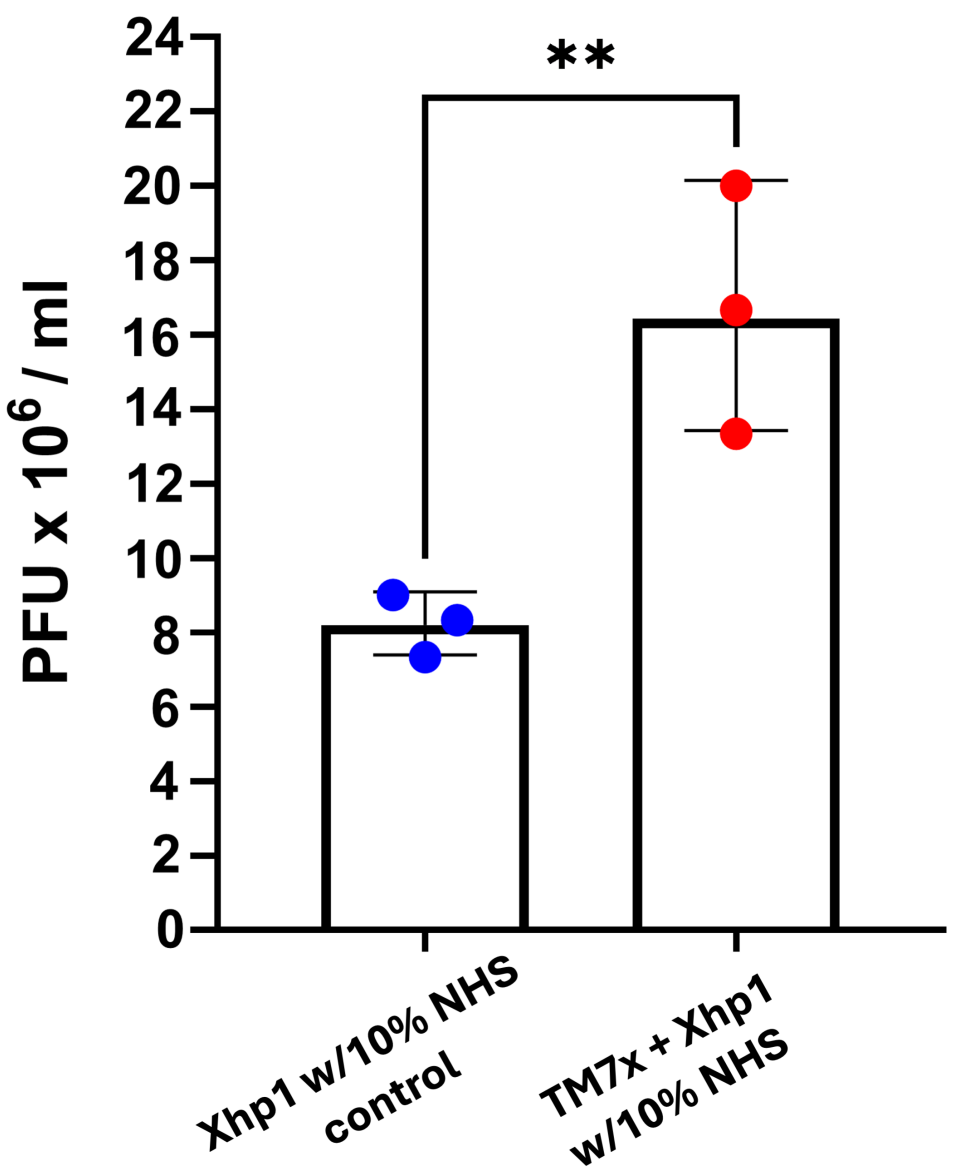

Fig. S5: PFU quantification of Xhp1 under different conditions. Xhp1 phage particles, mixed with or without isolated TM7x, were embedded in soft-agar droplets containing BHI supplemented with 10% heat-inactivated NHS. Following overnight incubation, phages were quantified by plaque assay (see Materials and Methods). Statistical analysis was performed through two-tailed unpaired Student's *t*-test. $P < 0.01$(**).

Table S1: Bacterial strains used in this study

Species and strain	Genotype and description	References or sources
Schaalia odontolytica strains<br>• XH001<br>• XH001ΔXhp1	Wild-type oral isolate<br>Prophage-free derivative of XH001	(6)<br>(22)
• *Actinomyces spp.* F0309	Wild-type oral isolate	BEI resources (NIAID,NIH)
• *Nanosynbacter lyticus* TM7x<br>• *Nanosynbacter spp*. BB006	Obligate symbiont of XH001<br>Obligate symbiont of F0309	(6)<br>(33)
Episymbiotic coculture<br>• XH001/TM7x<br>• XH001ΔXhp1/TM7x<br>• F0309/TM7x	TM7x forming episymbiosis with XH001<br>TM7x forming episymbiosis with prophage-free XH001<br>TM7x forming episymbiosis with F0309	(6)<br>This work<br>This work
Escherichia coli strain<br>• DH5α	Laboratory strain	ATCC

Table S2: Primers used in this study

Primers	Sequences
XH001 16s rRNA	**Forward** GCGGAGCATGCGGATTA<br>**Reverse** AACGTGCTGGCAACATAGGG
Phage lysin<br>(genome accession :MG941013.1, gene ID XHP1_00023)	**Forward** ACTGGGACGACGACAGTAT<br>**Reverse** CCGGACGACGTGTTGAAT
Phage capsid protein<br>(genome accession :MG941013.1, gene ID XHP1_00007)	**Forward** CGTCAAGGTCAAGGACTACAAG<br>**Reverse** AGTCGAAGGACTTCTCCTGAT
Phage tail protein<br>(genome accession :MG941013.1, gene ID XHP1_00013)	**Forward** CCCATCCGTAAGACCATTCA<br>**Reverse** GACCATCAGGAGACGGTATTC
Phage integrase<br>(genome accession :MG941013.1, gene ID XHP1_00029)	**Forward** TGCCTCATCCGATAGGTATGCGTCT<br>**Reverse** TAGCGAGGGCACCTGGGTCAGT

Table S3: Active lytic infection rate of Xhp1 against TM7x

Total TM7x cells analyzed	TM7x displaying intracellular Xhp1 replication (%)	TM7x displaying Xhp1-induced lysis (%)	Active lytic infection ratio (%)
**283**	**3** (1.1%)	**6** (2.1%)	**9** (3.2%)

Table S4: List of primers used for $GDPD_{Xhp1}$ protein cloning and expression

Primer name	5'-3'
GDP_up_fwd	GCACCATATGAGCCTGCGCGGAGCCTGG
GDP_up_rev	GATTTAAGCCCCGATGCAGAAGGCCCCACC
GDP_CatP_fwd	TCTGCATCGGGGCTTAAATCAAGGAAAAATCC
GDP_CatP_rev	GTGGTCGCCCTTTCTACGGGGTCTGACG
GDP_dwn_fwd	CCCGTAGAAAGGGCGACCACCTGCACGC
GDP_dwn_rev	GCTCGAATTCCGAAGCCGGCACCGTCAAG
GDP_PCWU3_fwd	GCCGGCTTCGGAATTCGAGCTCGGTACC
GDP_PCWU3_rev	CGCGCAGGCTCATATGGTGCACTCTCAG
GDP_up check	CGAGCTACCTCCGAAGAGCG
GDP_dwn_check	GCGCGACCATCATCACCG
GDPD V1_fwd	GCAAATGGGTGTGGATGAGCTGACAATCTATCG
GDPD V1_rev	CGGATCCGCGCTACGGATGCACGCGAGATG
pET28a_V1_fwd	GCATCCGTAGCGCGGATCCGAATTCGAG
pET28a_V1_rev	GCTCATCCACACCCATTTGCTGTCCACC
GDPD_F_Check	GACGTGATCGGTATGGAG
pET28_F_Check	GCTAGTTATTGCTCAGCG
